# Sentinel Node Mapping for Breast Cancer: Current Situation

**DOI:** 10.1155/2012/361341

**Published:** 2012-08-15

**Authors:** Sergi Vidal-Sicart, Renato Valdés Olmos

**Affiliations:** ^1^Nuclear Medicine Department, Hospital Clínic Barcelona, 08036 Barcelona, Spain; ^2^Nuclear Medicine Department, CRC-MAR, 08003 Barcelona, Spain; ^3^Nuclear Medicine Department, The Netherlands Cancer Institute, Antoni van Leeuwenhoek Hospital, 1066 CX Amsterdam, The Netherlands

## Abstract

Axillary node status is a major prognostic factor in early-stage disease. Traditional staging needs levels I and II axillary lymph node dissection. Axillary involvement is found in 10%–30% of patients with T1 (*<*2 cm) tumours. Sentinel lymph node biopsy is a minimal invasive method of checking the potential nodal involvement. It is based on the assumption of an orderly progression of lymph node invasion by metastatic cells from tumour site. Thus, when sentinel node is free of metastases the remaining nodes are free, too (with a false negative rate lesser than 5%). Moreover, Randomized trials demonstrated a marked reduction of complications associated with the sentinel lymph node biopsy when compared with axillary lymph node dissection. Currently, the sentinel node biopsy procedure is recognized as the standard treatment for stages I and II. In these stages, this approach has a positive node rate similar to those observed after lymphadenectomy, a significant decrease in morbidity and similar nodal relapse rates at 5 years. In this review, the indications and contraindications of the sentinel node biopsy are summarized and the methodological aspects discussed. Finally, the new technologic and histologic developments allow to develop a more accurate and refinate technique that can achieve virtually the identification of 100% of sentinel nodes and reduce the false negative rate.

## 1. Introduction

Breast cancer is the most common cancer in women worldwide. In 2010, it was estimated that in the USA there were nearly 210,000 new cases of invasive breast cancer and more than 40,000 deaths. Axillary node status is a major prognostic factor in early-stage disease, and this information is important for treatment. Traditional staging needs levels I and II axillary lymph node dissection. Axillary involvement is found in 10%–30% of patients with T1 (<2 cm) tumours. This rate reaches 45% for small T2 tumours (2.1–3 cm) and 55%–70% for larger tumours (>3 cm). Routine axillary lymphadenectomy adds the risk of lymphedema, sensory disturbances, and chronic pain.

Sentinel lymph node biopsy is a minimal invasive method of checking the potential nodal involvement. It is based on the assumption of an orderly progression of lymph node invasion by metastatic cells from tumour site. Thus, the nodal basin is free of malignancy if the sentinel lymph node is not involved. Patients with metastasis to a sentinel node would undergo either immediate or delayed completion lymph node dissection. Randomized trials demonstrated a marked reduction of complications associated with the sentinel lymph node biopsy when compared with axillary lymph node dissection. In the ALMANAC trial, more than 1,000 patients were randomized to undergo either axillary lymphadenectomy or sentinel node biopsy. Lymphedema was present in 13% of the axillary lymphadenectomy group and in 5% of the sentinel node group 12 months after surgery.

In 2005, guidelines from the American Society of Clinical Oncology stressed that a multidisciplinary team should aim at a sentinel node identification rate of 85% with a false-negative rate of 5% or less in order to abandon axillary dissection. False-negative rate is the proportion of axillary node dissection, positive cases with a negative sentinel node at biopsy. False-negative cases may result from massive involvement of the real sentinel node, a circumstance that interferes with the uptake of both radiocolloid and dye and lymph flow that goes to a node other than the true sentinel node [1]. 

A meta-analysis of 69 trials with a total of 8,059 patients in whom sentinel node biopsy was followed by axillary dissection showed substantial variability in the performance of the technique throughout different centers. However, recent results from large multi-institutional trials showed that all have achieved excellent identification rates, ranging from 93% to 97%, but that none achieved a false-negative rate lower than 5%. The lowest false-negative rates were obtained in the 2 studies in which preoperative lymphoscintigraphy and dual mapping during surgery were required. In the ALMANAC trial, the false-negative rate was 6.7%. However, if only blue sentinel nodes are considered, the false-negative rate was 9.1% [2–5].

## 2. Clinical Scenarios and Current Indications

Clinical indications for this approach have been changing through the years and there is still a debate on some of them. Many centers use sentinel node biopsy only in patients with a unifocal tumour smaller than 3 cm, whereas others have extended the application to patients with large T2 or T3 (>5 cm) tumours, multifocal/multicentric carcinomas, or to patients who have received neoadjuvant chemotherapy.

Currently, the sentinel node biopsy procedure is recognized as the standard treatment for stages I and II. In these stages, this approach has a positive node rate similar to those observed after lymphadenectomy, a significant decrease in morbidity and similar nodal relapse rates at 5 years. No significant differences on disease-free survival, overall survival, and local control of disease were seen in case of negative sentinel node [5]. The indications and recommendations of the sentinel node biopsy are summarized in Table 1.

(i) Pregnancy is no contraindication for sentinel node biopsy, but only for blue dye, and it has been demonstrated that the dose to the foetus from this procedure is negligible [6].

(ii) The evidence regarding the safety of sentinel node biopsy is mainly based on studies including T1 and small T2 tumours only. However, in patients with larger tumours (T3-T4), the false negative rate has been similar and no increased axillary recurrence has been reported [6, 7]. 

(iii) Multifocal breast cancer is defined as separate foci of ductal carcinoma more than 2 cm apart within the same quadrant, while multicentric breast cancer indicates the presence of separate independent foci of carcinoma in different quadrants. The prevalence of axillary metastases and false negative results is higher in multifocal or multicentric tumours. However, the reported axillary recurrence rates are acceptable also in patients with multifocal or multicentric tumours [6–9]. 

(iv) DCIS does not metastasize to regional lymph nodes. However, invasion is missed in up to 40% of patients in the preoperative diagnosis. Therefore, sentinel node biopsy is recommended in patients undergoing mastectomy. In patients with breast conservation, sentinel lymph node biopsy can be performed as a second operation if invasion is detected in the surgical specimen [6, 10].

(v) Palpable axillary nodes may be tumour negative in up to 40% of the patients. Preoperative axillary ultrasound with fine needle aspiration cytology or core needle biopsy from the suspicious nodes is a widely accepted policy. In many units, sentinel node biopsy is performed also in patients with palpable nodes if negative in the preoperative diagnosis [6]. 

(vi) Internal mammary sentinel node detection rate is significantly affected by the depth of radiopharmaceutical injection. It is generally recognized that mapping of inner mammary chain requires deep injection (peritumoural or intratumoural) of radiotracer. With this approach, internal mammary chain sentinel nodes have been detected in about 30% of patients with breast cancer, of which about 60%–90% could be harvested during surgery and 11%–27% of them will have metastases. However, the significance of internal mammary sentinel node biopsy is under debate. There is evidence that mapping it leads to stage migration and modification of treatment planning with respect to radiotherapy and systemic therapy, but more evidence is necessary to support that it will improve the outcome of treatment and survival [11, 12].

A second sentinel node biopsy may be performed in patients with a local recurrence after breast conservation and negative axillary sentinel node biopsy. The success rate may be lower when compared with a primary sentinel node biopsy. Furthermore, extra axillary sentinel nodes are visualized more frequently. Sentinel node biopsy can be performed in patients undergoing breast surgery due to a local recurrence after breast conservation in DCIS. Furthermore, plastic surgery with breast augmentation or reduction does not contraindicate the procedure. In prior excisional biopsy the lymph drainage is probably changed in patients who have undergone previous breast surgery (oncologic and nononcologic). Extra-axillary drainage is identified more frequently in reoperative sentinel node biopsy than in former sentinel node biopsy. However, there are evidences that sentinel node biopsy performed in the area of previous breast biopsy do not affect the accuracy of the procedure [6, 13].

(vii) Before neoadjuvant chemotherapy, sentinel node biopsy gives a more precise axillary staging, with more information about the nodal spread. But it may delay the beginning of the therapy, and two surgeries can be necessary. After neoadjuvant chemotherapy, the sentinel node biopsy may lead to an underestimation of the initial stage. On the other hand, axillary nodal status after neoadjuvant therapy is also a highly significant prognostic factor. Pathologic complete response in the axilla can be achieved in up to 40% of the patients. These patients avoid axillary lymph node dissection and associated morbidity. Available data show that there are no significant differences in the success rate of sentinel node biopsy according to clinical tumour size or clinical nodal status, and that the false-negative rate is not affected by tumour response to chemotherapy [14, 15]. 

Despite this, the current controversy in this scenario lies on the question of axillary lymph node dissection after a positive sentinel node biopsy, mainly stimulated by the recent publication of the ACOSOG-Z0011 data [16].

## 3. Methodological Aspects

The sentinel node procedure uses a radiotracer, a blue dye, or both to find the node. Radiopharmaceuticals for sentinel lymph node technique are colloids labelled with ^99m^Tc. These colloids allow sentinel node visualization with a gamma camera before surgery and intraoperative detection with a hand-held gamma probe. Controversies exist with regard to the selection of agents, the size of the particles of the radiotracer, the optimal route for injection, time to scintigraphy and intraoperative detection, and whether or not extra-axillary lymph nodes should be considered as well.

Mariani et al. suggested that ^99m^Tc-labeled colloids with most of the particles in the 100 to 200 nm size range would be ideal for sentinel node biopsy in breast cancer. The choice of tracer is often guided by local availability. ^99m^Tc-labeled colloids of human serum albumin are often used in Europe, ^99m^Tc-sulfur colloid is used in the United States (sometimes after filtration through a 0.1 or 0.2 mm membrane), and ^99m^Tc-antimony trisulfide in Australia. There is no established difference between a 1-day protocol (same-day imaging and surgery) and a 2-day protocol [6].

Lymphatic drainage of the breast is not completely understood. After an experience of almost 20 years, it is generally assumed that both deep and superficial injection approaches are valid techniques and may be complementary. In most early sentinel node studies, the tracer was injected around the tumour and such peritumoral injection was considered the gold standard against which all other mapping techniques were tested. Many investigators have reported good results using injection into the breast skin over the tumour or using a periareolar, subareolar, or even intratumoural injection. One clearly established advantage of deep injections (peritumoral/intratumoural) is its ability to also reveal extra-axillary drainage. On the other hand, superficial injection techniques (subdermal/areolar) provide a faster lymphatic drainage, yield more radioactive counts at the axillary sentinel nodes, and are independent of the palpable or nonpalpable nature of the tumour. Hence, the tracer is not always transported to the same axillary node, regardless the injection site. However, if the goal is axillary staging only, a superficial tracer injection is preferable due to better visualization of axillary sentinel nodes. If the aim is to stage also the extra-axillary nodal basins, peri- or intratumoural injection should be applied [6, 17, 18].

Lymphoscintigraphy has been an essential component for the preoperative sentinel node identification in breast cancer. Lymphoscintigraphy has the potential to both improve accuracy and reduce morbidity relative to gamma probe alone by providing the surgeon with a roadmap of lymphatic drainage and the location of sentinel nodes [6]. 

**Figure 1:**
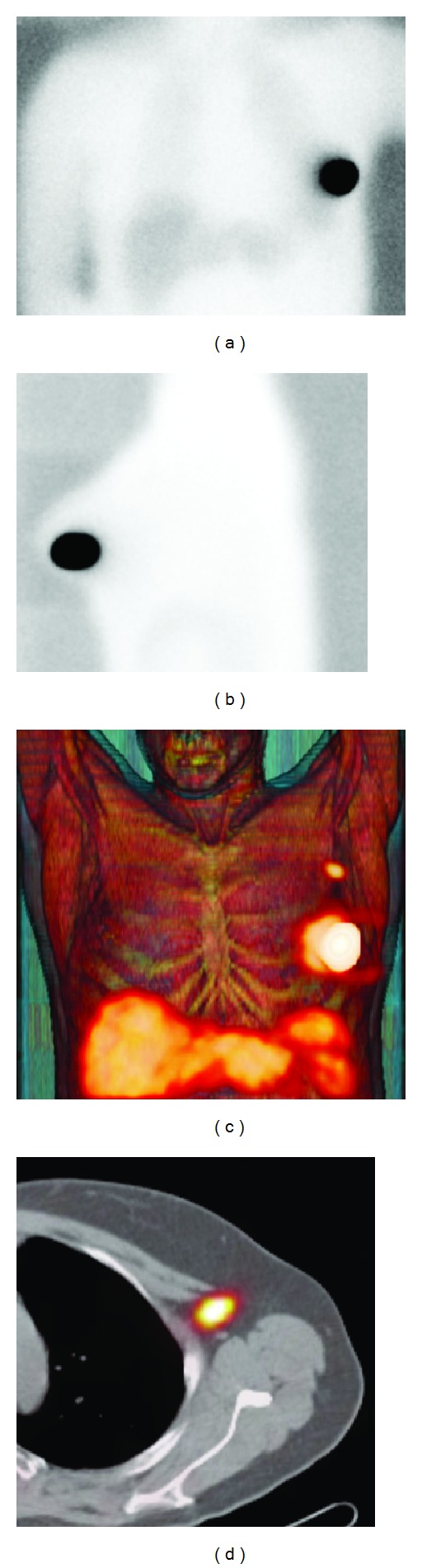
Planar images (a)-(b) showing no drainage of ^99m^Tc-nanocolloid from the injection site in left breast. By contrast, fused SPECT/CT with volume rendering (c) shows drainage to the left axilla with one sentinel node in level 1 (d).

To identify all sentinel nodes and avoid confusion with a stasis in a lymphatic vessel, images are acquired with an adequate delay after injection. Lymphatic drainage can be slower in old or overweight patients. With planar scintigraphy, combining 2 views may help prevent some sentinel nodes from being missed (Figure 1).

**Figure 2:**
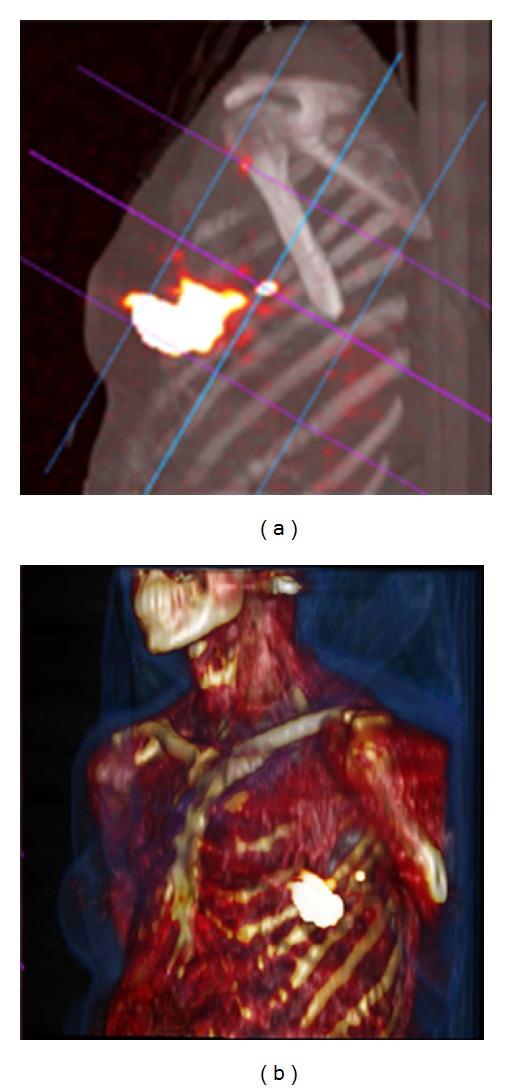
Fused SPECT/CT (a) displayed with maximum intensity projection (MIP) showing a sentinel node in the left axilla. SPECT/CT with volume rendering for 3D display (b) adds an excellent overview for surgical planning.

The advent of SPECT/CT reinforces the potential of preoperative lymphoscintigraphy. The functional information from SPECT can be combined with the morphological information from CT by applying both techniques in one session. The resulting SPECT/CT fused images depict sentinel nodes in an anatomical landscape providing a helpful roadmap for surgeons. In recent years, SPECT/CT has been used in breast cancer patients with unusual or complex drainage. This is the case in patients with drainage outside the axilla. SPECT/CT can also detect hot nodes missed by planar imaging because of shine-through from the injection site or in overweight patients [19, 20] (Figure 2). 

Differentiating a true sentinel node from a secondary echelon node is difficult. Also, lymphatics of a tumor site can drain simultaneously to more than one sentinel node. 

Lymphoscintigraphy is able to identify sentinel nodes in a majority of cases by acquiring early and delayed planar images. In current protocols, SPECT/CT is performed following delayed planar images (mostly 2–4 hours after tracer administration). This sequential acquisition is helpful to clarify the role of both modalities. However, it is necessary to specify the criteria for sentinel node identification on preoperative images. Major criteria to identify lymph nodes as sentinel nodes are the visualization of lymphatic ducts, the time of appearance, the lymph node basin, and the intensity of lymph node uptake. Following these criteria visualized radioactive lymph nodes may be classified as follows

- Definitively sentinel nodes: this category concerns all lymph nodes draining from the site of the primary tumour through an own lymphatic vessel, or a single radioactive lymph node in a lymph node basin.
- Highly probable sentinel nodes: this category includes lymph nodes appearing between the injection site and a first draining node, or nodes with increasing uptake appearing in other lymph node stations
- Less probable sentinel nodes: all higher echelon nodes may be included in this category.

The use of these categories to characterize radioactive lymph nodes is also helpful for clinical decision making. Lymph nodes of the first two categories (definitively sentinel node or highly probable sentinel node) are the nodes recognized by the nuclear physician and that must be removed at the operation room by the surgeon. Less probable sentinel nodes may sometimes be removed depending on the degree of remaining radioactivity measured by the gamma probe during the control of the surgical field [21].

Dyes cause the blue colouring as they pass slowly through the sentinel node. Isosulfan blue is of greater use in the United States, and patent blue V in Europe. Data from NSABP B-32 and ACOSOG-Z0010 with isosulfan, and from ALMANAC with patent blue V, showed that the overall risk of allergic reaction is close to 1% for both dyes, with an approximately 0.1% risk of severe reactions (grade III). Despite a risk of allergic reactions to blue dye, most teams favour the combinative mapping procedure [6].

In one multicentric study, the false-negative rate was 17.7% if only 1 node was resected, 10% if 2, 6.9% if 3, 5.5% if 4, and 1% if 5 or more. These results should not imply removal of multiple nodes for an optimal sentinel node procedure. However, all identified hot or blue nodes should be resected. Careful palpation by the surgeon of the operative field is also required to identify any suggestive large, hard nonblue and nonradioactive nodes [5].

## 4. New Developments

During the last decade, intraoperative imaging devices have become available for clinical practice and can be used during surgery as they provide information that can be combined with data obtained with conventional gamma probes. However, since nonimaging probes are still the standard equipment for detection of radiolabeled tissue in the operating room, the role of intraoperative imaging is generally limited and constitutes an additional aid to the surgeon. Intraoperative imaging with portable gamma cameras provides real-time imaging with a global overview of all radioactive hot spots in the whole surgical field. Its position can be adjusted to also show sentinel nodes near the injection area, which can easily be missed when using the non-imaging probe.

Some authors have tried to clarify the added value of portable gamma camera in clinical practice. In fact, there is no consensus on the real need for an intraoperative imaging device to help detection of the sentinel lymph node. The usefulness of the portable gamma cameras in breast cancer patients lies on when no conventional gamma camera is available or in particular cases with extra-axillary drainage (intramammary and internal mammary chain nodes) or when the sentinel lymph node is located very close to the injection site. Although the majority of these cases can be solved with the presurgical information provided by SPECT/CT, real-time images acquired with a portable gamma camera can be an alternative to hybrid imaging. On the other hand, the preoperative anatomical information obtained by SPECT/CT appears to lead to a more optimal use of portable devices for sentinel localization in the operation room. Using an intraoperative imaging device implies the possibility to better planning the procedure and to monitor the lymphatic basin before and after removal of the hot nodes, so to verify completeness of lymph node excision. After excision of each lymph node, a new image is acquired and compared with the situation before excision. If focal radioactivity remains at the same location, it is concluded that another possible sentinel lymph node is still in place [22, 23].

Thanks to novel technological possibilities, combining a spatial localization system and two tracking targets to be fixed on a conventional, hand-held gamma probe results in new 3D visualization of the traditional acoustic signal of the gamma probe. In this regard, the most recent development is the system so-called free-hand SPECT, in which a continuous positioning system installed in the operating room is based on a fix-pointing device, on the patient's body and, respectively, on the hand-held gamma counting probe, thus permitting a virtual reconstruction in a 3D environment. The surgeon can easily check location and depth of the foci of radioactivity accumulation to be resected, and this 3D information may be further used for precise localization and targeting of the radioactive sentinel lymph node(s) and of tumour tissue. The device can ensure permanent assistance and transparent documentation of soft tissue removal during the intervention [24].

**Figure 3:**
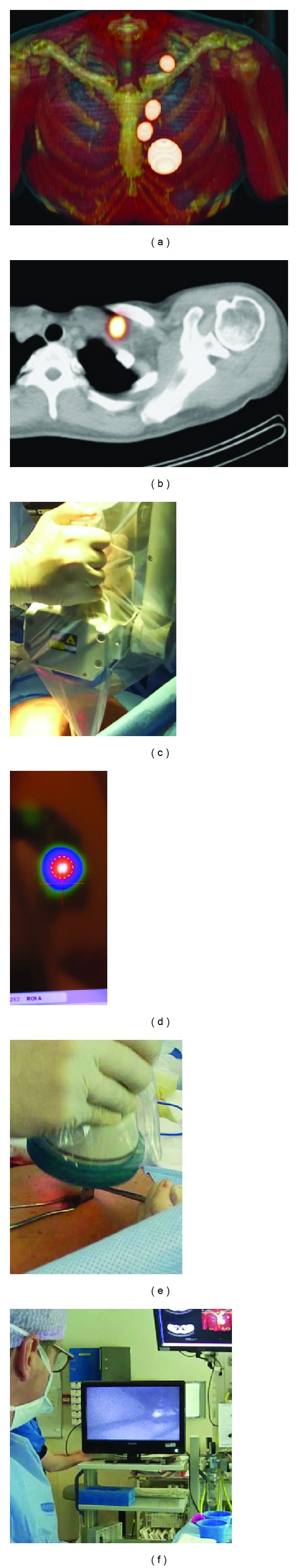
Fused SPECT/CT displayed with volume rendering (a) showing drainage of ICG/^99m^Tc-nanocolloid to the internal mammary chain and left supraclavicular area. The supraclavicular node is shown on axial SPECT/CT (b). The inferior internal mammary node as well as the supraclavicular one were removed using a portable gamma camera (c) to detect the radioactive signal (d) and a fluorescence camera (e) to localize the fluorescent sentinel node (f).

On the other hand, the possibility of combining the current radiopharmaceuticals with other agents opens new fields to explore. In this regard, a radiolabeled nanocolloid agent has been combined with ICG, a fluorescent agent, for sentinel node detection. In contrast to the use of a single-fluorescent agent, this bimodal tracer may allow the surgeons to integrate the standard approach based on radioguided detection with a portable gamma camera with a new optical modality based on fluorescent signal detection. This approach is being successfully applied in several malignancies and to localize sentinel nodes outside the axilla in breast cancer (Figure 3) [25].

For all these new intraoperative modalities, the preoperative anatomical SPECT/CT acquisition remains essential and is the starting point for surgical planning.

Before sending for histological examination, any lymph node removed should be rechecked by the probe to demonstrate that they are radioactive. Histopathological assessment of the sentinel lymph node is the golden standard procedure for the subsequent management of the conservative surgery in breast cancer patients. However, this “golden standard” is highly variable between centres. In many units, the sentinel nodes are assessed intraoperatively using imprint cytology, frozen sectioning, or both, and more thoroughly after the operation. The sensitivity of the intraoperative diagnosis in variable and many units do not adopt it at all. Some molecular methods have been used previously for sentinel node diagnosis but have shown a lack of reproducibility, a longer time for the intraoperative assessment, and an inability to study the whole lymph node. A new molecular method has been developed recently, based on an one-step nucleic acid amplification (OSNA) method. This procedure is in the phase of validation in many centres, although there are others that routinely apply this method [26].

In summary, after two decades of sentinel lymph node biopsy use in breast cancer, this technique is the current standard of care for locoregional staging. However, some concerns remain as there is no only one standardized technique and many controversies are still unsolved. However, with the recent technologic and histologic developments, a more accurate and refinate technique can be achieved by virtually identifying-100% of sentinel nodes and reduce the false negative rate.

## Figures and Tables

**Table 1:** Recommendations for SLN biopsy.

Clinical scenario	Indication of sentinel node biopsy
T1 or T2 tumours	Established
Older age	Established
Obesity	Established
Before preoperative systemic therapy	Established
Male breast cancer	Established
DCIS with mastectomy	Established
Internal mammary chain	Established but controversial
DCIS without mastectomy	Controversial, except for DCIS with suspected or proven microinvasion
Pregnancy	Controversial
Suspicious, palpable axillary nodes	Controversial
T3 or T4 tumours	Controversial
Multicentric or multifocal tumours	Controversial
Prior diagnostic or excisional breast biopsy	Controversial
Prior axillary surgery	Controversial
Prior non-oncologic breast surgery	Controversial
After preoperative systemic therapy	Controversial
Inflammatory breast cancer	Not recommended

DCIS: ductal carcinoma in situ.

Controversial indications suggest that the indication is not universally accepted or the evidence behind the practice is limited.

## References

[1] Lyman GH, Giuliano AE, Somerfield MR (2005). American Society of Clinical Oncology guideline recommendations for sentinel lymph node biopsy in early-stage breast cancer. *Journal of Clinical Oncology*.

[2] Kim T, Giuliano AE, Lyman GH (2006). Lymphatic mapping and sentinel lymph node biopsy in early-stage breast carcinoma: a metaanalysis. *Cancer*.

[3] Gill G (2009). Sentinel-lymph-node-based management or routine axillary clearance? One-year outcomes of sentinel node biopsy versus axillary clearance (SNAC): a randomized controlled surgical trial. *Annals of Surgical Oncology*.

[4] Goyal A, Newcombe RG, Chhabra A, Mansel RE (2006). Factors affecting failed localisation and false-negative rates of sentinel node biopsy in breast cancer—results of the ALMANAC validation phase. *Breast Cancer Research and Treatment*.

[5] Krag DN, Anderson SJ, Julian TB (2007). Technical outcomes of sentinel-lymph-node resection and conventional axillary-lymph-node dissection in patients with clinically node-negative breast cancer: results from the NSABP B-32 randomised phase III trial. *The Lancet Oncology*.

[6] Cheng G, Kurita S, Torigian DA, Alavi A (2011). Current status of sentinel lymph-node biopsy in patients with breast cancer. *European Journal of Nuclear Medicine and Molecular Imaging*.

[7] Meretoja TJ, Leidenius MH, Heikkilä PS, Joensuu H (2009). Sentinel node biopsy in breast cancer patients with large or multifocal tumors. *Annals of Surgical Oncology*.

[8] Spillane AJ, Brennan ME (2011). Accuracy of sentinel lymph node biopsy in large and multifocal/multicentric breast carcinoma—a systematic review. *European Journal of Surgical Oncology*.

[9] Brouwer OR, Vermeeren L, van der Ploeg IM (2012). Lymphoscintigraphy and SPECT/CT in multicentric and multifocal breast cancer: does each tumour have a separate drainage pattern?: results of a Dutch multicentre study (MULTISENT). *European Journal of Nuclear Medicine and Molecular Imaging*.

[10] Meretoja TJ, Heikkilä PS, Salmenkivi K (2012). Outcome of patients with ductal carcinoma in situ and sentinel node biopsy. *Annals of Surgical Oncology*.

[11] Paredes P, Vidal-Sicart S, Zanón G (2005). Clinical relevance of sentinel lymph nodes in the internal mammary chain in breast cancer patients. *European Journal of Nuclear Medicine and Molecular Imaging*.

[12] Bourre JC, Payan R, Collomb D (2009). Can the sentinel lymph node technique affect decisions to offer internal mammary chain irradiation?. *European Journal of Nuclear Medicine and Molecular Imaging*.

[13] Rodriguez Fernandez J, Martella S, Trifirò G (2009). Sentinel node biopsy in patients with previous breast aesthetic surgery. *Annals of Surgical Oncology*.

[14] Kelly AM, Dwamena B, Cronin P, Carlos RC (2009). Breast cancer sentinel node identification and classification after neoadjuvant chemotherapy -systematic review and metaanalysis. *Academic Radiology*.

[15] Pinero A, Giménez J, Vidal-Sicart S, Intra M (2010). Selective sentinel lymph node biopsy and primary systemic therapy in breast cancer. *Tumori*.

[16] Giuliano AE, Hunt KK, Ballman KV (2011). Axillary dissection vs no axillary dissection in women with invasive breast cancer and sentinel node metastasis: a randomized clinical trial. *Journal of the American Medical Association*.

[17] Estourgie SH, Nieweg OE, Valdés Olmos RA, Rutgers EJT, Kroon BBR (2004). Lymphatic drainage patterns from the breast. *Annals of Surgery*.

[18] Noguchi M, Inokuchi M, Zen Y (2009). Complement of peritumoral and subareolar injection in breast cancer sentinel lymph node biopsy. *Journal of Surgical Oncology*.

[19] Van Der Ploeg IMC, Valdés Olmos RA, Nieweg OE, Rutgers EJT, Kroon BBR, Hoefnagel CA (2007). The additional value of SPECT/CT in lymphatic mapping in breast cancer and melanoma. *Journal of Nuclear Medicine*.

[20] Vermeeren L, Van Der Ploeg IMC, Valdés Olmos RA (2010). SPECT/CT for preoperative sentinel node localization. *Journal of Surgical Oncology*.

[21] Vidal-Sicart S, Roberto Brouwer O, Valdés-Olmos RA (2011). Evaluation of the sentinel lymph node combining SPECT/CT with the planar image and its importance for the surgical act. *Revista Espanola de Medicina Nuclear*.

[22] Valdés Olmos RA, Vidal-Sicart S, Nieweg OE (2009). SPECT-CT and real-time intraoperative imaging: new tools for sentinel node localization and radioguided surgery?. *European Journal of Nuclear Medicine and Molecular Imaging*.

[23] Vidal-Sicart S, Paredes P, Zanón G (2010). Added value of intraoperative real-time imaging in searches for difficult-to-locate sentinel nodes. *Journal of Nuclear Medicine*.

[24] Wendler T, Herrmann K, Schnelzer A (2010). First demonstration of 3-D lymphatic mapping in breast cancer using freehand SPECT. *European Journal of Nuclear Medicine and Molecular Imaging*.

[25] Brouwer OR, Buckle T, Vermeeren L (2012). Comparing the hybrid fluorescent-radioactive tracer Indocyanine green-99mTc-Nanocolloid with 99mTc-Nanocolloid for sentinel node identification: a validation study using lymphoscintigraphy and SPECT/CT. *Journal of Nuclear Medicine*.

[26] Bernet L, Cano R, Martinez M (2011). Diagnosis of the sentinel lymph node in breast cancer: a reproducible molecular method: a multicentric Spanish study. *Histopathology*.

